# Comparative evaluation of *Sm1-7*-qPCR systems incorporated with Maxwell kit-based and NaOH-based DNA extraction methods for the detection of *Schistosoma mansoni* infection

**DOI:** 10.1186/s40249-026-01444-7

**Published:** 2026-05-11

**Authors:** Yi Mu, Thomas G. Egwang, Candia Rowel, Moses Adriko, Malcolm K. Jones, Helena Ullyartha Pangaribuan, Toni Wandra, Donald P. McManus, Pengfei Cai

**Affiliations:** 1 Molecular Parasitology Laboratory, QIMR Berghofer, Brisbane, Australia; 2 Department of Immunology and Parasitology, Med Biotech Laboratories, Kampala, Uganda; 3 Vector Borne & Neglected Tropical Disease Control Division, Ministry of Health, Kampala, Uganda; 4 Centre for Tropical Health and Emerging Diseases, QIMR Berghofer, Brisbane, Australia; 5 School of Veterinary Science, The University of Queensland, Brisbane, Australia; 6 Center for Biomedical Research, National Research and Innovation Agency (BRIN), Research Organization for Health, Bogor, Indonesia; 7 Directorate of Postgraduate, Sari Mutiara Indonesia University, Medan, Indonesia; 8 School of Biomedical Sciences, The University of Queensland, Brisbane, Australia

**Keywords:** *Schistosoma mansoni*, *Sm1-7-*qPCR, NaOH-based DNA extraction, Real-time PCR, Molecular diagnosis, Uganda

## Abstract

**Background:**

The World Health Organization’s latest roadmap for neglected tropical diseases (NTDs) 2021–2030 has highlighted diagnostics as one of four focus areas to achieve the targets set for 2030. Molecular diagnostic tests, such as quantitative real-time PCR (qPCR), are valuable for diagnosing NTDs, including schistosomiasis. This study aimed to comparatively evaluate two *Sm1-7*-qPCR systems incorporating different DNA extraction approaches for the detection of *Schistosoma mansoni* infection.

**Methods:**

Two copro-DNA-qPCR systems were developed: a Maxwell kit-based copro-DNA-*Sm1-7*-qPCR system (*Sm1-7*-qPCR-S1) and a NaOH-based copro-DNA-*Sm1-7*-qPCR system (*Sm1-7*-qPCR-S2). Both systems were tested on faecal samples collected from schistosomiasis endemic and non-endemic areas in Uganda (*n* = 482). A composite reference based on positive results of either the Kato-Katz (KK) or qPCR assays was used for calibration. The *S. mansoni* positivity rates were analyzed based on the results of different diagnostics. Differences in DNA concentration and cycle threshold (Ct) values from the two copro-DNA-qPCR systems were compared (Wilcoxon signed-rank test).

**Results:**

The overall *S. mansoni* positivity rate was 52.9% (229/433) according to the composite reference, whereas the KK, *Sm1-7*-qPCR-S1 and *Sm1*-*7*-qPCR-S2 recorded positivity rates of 36.7% (159/433), 43.9% (190/433), and 47.6% (206/433), respectively. *Sm1-7*-qPCR-S2 demonstrated a sensitivity of 90.0%, outperforming the KK and *Sm1-7*-qPCR-S1 with sensitivities of 69.4% and 83.0%, respectively (*P* < 0.0001 and *P* = 0.015, respectively, *χ*^2^_m_ test). For samples positive in both *Sm1-7*-qPCR systems (*n* = 179), Ct-values obtained from *Sm1-7*-qPCR-S2 were significantly lower than those from *Sm1-7*-qPCR-S1 (*P* < 0.0001, Wilcoxon signed-rank test). A significant inverse correlation between Ct values and KK faecal egg counts was observed for both systems (*Sm1-7*-qPCR-S1, r = −0.5637, *P* < 0.0001; *Sm1*-*7*-qPCR-S2, r = −0.5723, *P* < 0.0001). The KK method showed substantial agreement with the composite reference (*ƙ* = 0.682), while both *Sm1*-*7*-qPCR systems exhibited almost perfect agreement with the reference (*ƙ* = 0.821 and 0.894, respectively).

**Conclusions:**

This study highlights that DNA samples extracted from both systems are suitable for robust qPCR amplification. The *Sm1-7*-qPCR-S2 system offers several advantages over the *Sm1-7*-qPCR-S1 system, including improved sensitivity, field accessibility, and lower cost. With further refinement, the *Sm1-7*-qPCR-S2 system could be integrated into the existing schistosomiasis surveillance network to aid the elimination of the disease.

**Graphical Abstract:**

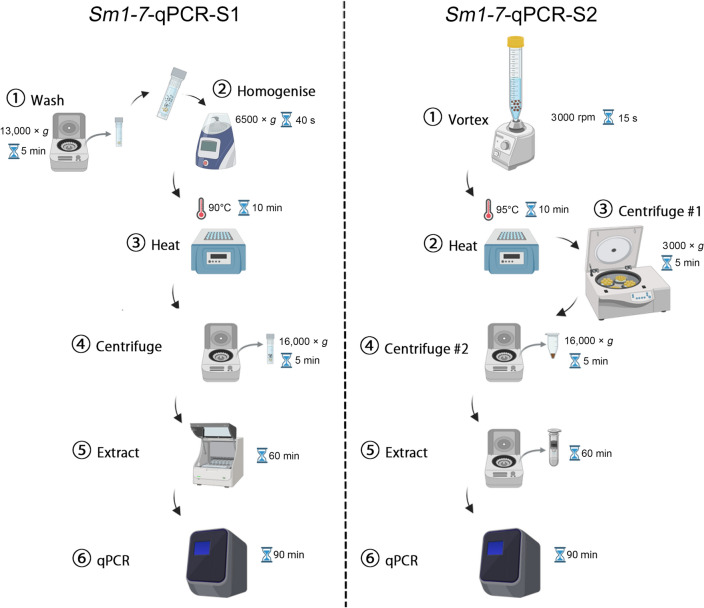

**Supplementary Information:**

The online version contains supplementary material available at 10.1186/s40249-026-01444-7.

## Introduction

### Background

Schistosomiasis is a severely debilitating and potentially fatal disease that afflicts more than 250 million people worldwide, with over 90% of cases in sub-Saharan Africa [1, 2]. It causes chronic malnutrition, stunted growth, cognitive impairment, and anemia, representing one of the major neglected tropical diseases (NTDs). In Uganda, *Schistosoma mansoni* and *S. haematobium* are prevalent in 95 and 4 districts out of the total 146 districts, respectively [3]. The Uganda NTD master plan 2023 estimates that 7.4 million individuals are currently infected with schistosomiasis, with 15.7 million at risk [4]. Despite significant progress, the disease remains a significant public health concern in the country, especially in communities located near major water bodies such as Lake Victoria [5].

For the diagnosis of *S. mansoni*, the WHO recommends using the Kato-Katz (KK) test to detect eggs in stool samples by microscopy. However, the KK method is labor-intensive and lacks sensitivity in detecting light infections, potentially leading to misdiagnosis in low-endemic areas. The WHO also suggests using the point-of-care circulating cathodic antigen (POC-CCA) test for *S. mansoni* diagnosis, but this assay has limitations, such as cross-reactivity, low specificity, and a ‘Trace’ (residual reactivity) reading issue, i.e., when trace results are interpreted as negative, active *Schistosoma* infection is frequently missed in many cases. However, considering “trace” as positive leads to a significant overestimation of infection rates in areas with low prevalence [6–8]. Molecular diagnostics based on nucleic acid amplification tests (NAATs) have emerged as powerful alternatives to conventional parasitological and antigen-based methods [9–14]. Conventional PCR assays provide qualitative detection of parasite DNA, whereas quantitative real-time PCR (qPCR) enables real-time detection, quantification of parasite burden, and indirect estimation of infection intensity, which are critical for surveillance and programmatic decision-making [15, 16]. Among these, the *Sm1-7*-qPCR assay, targeting a highly repetitive 121-bp tandem repeat sequence of *S. mansoni*, has demonstrated considerable sensitivity and specificity, particularly in low-endemic and non-endemic settings [17–19].

Despite their diagnostic advantages, the broader implementation of qPCR-based assays in endemic settings is often limited by DNA extraction costs, infrastructure requirements, and processing complexity. Simplified, low-cost extraction methods that maintain analytical performance are therefore essential to bridge the gap between diagnostic accuracy and field applicability. In this study, we developed and comparatively evaluated two *Sm1-7*-qPCR systems incorporating different DNA extraction strategies: a commercial Maxwell kit-based approach and a simplified NaOH-based method. The objectives are to (i) compare the diagnostic performance of both systems, (ii) assess differences in DNA yield and qPCR amplification efficiency, and (iii) evaluate the operational suitability of a NaOH-based extraction method for large-scale surveillance and elimination programs. The performance of both systems was assessed using faecal samples collected from schistosomiasis-endemic and non-endemic areas in Uganda.

## Methods

### Study population and sampling

The appropriate sample size was calculated using the Thrusfield formula [20], with an expected average *S. mansoni* prevalence of 50%, a 95% confidence interval (*CI*) and a 5% absolute error.

$$N = \frac{{1.96^{2} \times P_{\rm{exp}} \times (1 - P_{\rm{exp}} )}}{{d^{2} }}$$

*N*: total sample size; *P*_exp_: expected prevalence; *d*: absolute precision.

**Fig. 1:**
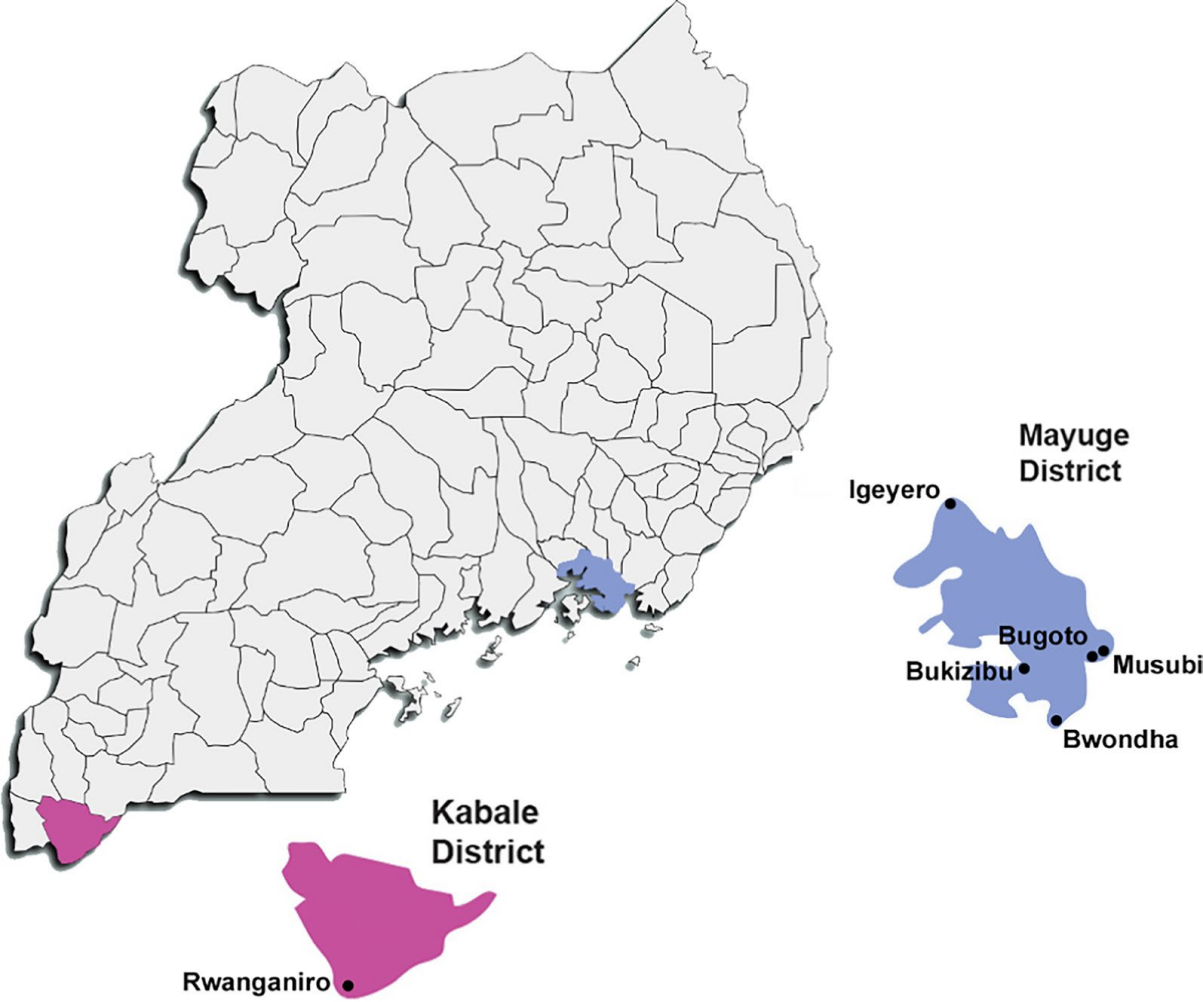
Illustration of the location of study sites. The study was conducted in five villages (Igeyero, Bugoto, Musubi, Bukizibu, and Bwondha) in Mayuge District and one village (Rwanganiro) in Kabale District, Uganda (created by paintmaps.com)

Based on this calculation, the estimated minimum sample size required was 384. The leaders of the communities and educators in the schools in the sampled areas were informed about the study. Information about the study was provided to individuals who expressed interest in participating. Participants had to be over 5 years old and agree to provide two stool samples. A total of 450 participants from five schistosomiasis-endemic villages [Igeyero (*n* = 100), Bugoto (*n* = 100), Musubi (*n* = 100), Bwondha (*n* = 100), and Bukizibu (*n* = 50)] in Mayuge District and 50 participants from a non-endemic village (Rwanganiro) in Kabale District were recruited between September and October 2022 (Fig. 1) [21–23]. Each individual provided two stool samples over a two-day period, with most samples collected in the early morning. The KK technique was used to detect parasite eggs in the stool samples [21]. For each stool sample, three KK slides were prepared by laboratory staff and examined by trained microscopists using a light microscope at Med Biotech Laboratories in Kampala, Uganda. The KK results were expressed as the number of eggs per gram (EPG) of faeces, calculated by multiplying the average number of eggs present in 6 smears by 24 (Additional file 1: Table S1). To ensure the accuracy of the KK results, 10% of slides were randomly re-examined by an experienced microscopist. The first-day stool samples were shipped on dry ice to the QIMR Berghofer in Australia for molecular diagnosis.

### Maxwell kit-based DNA extraction

Faecal DNA was extracted using the Maxwell 16 LEV Plant DNA kit following a previously described method [13]. Briefly, 200 mg of faecal sample was mixed with 500 µl of ddH_2_O, and centrifuged at 13,000 × *g* for 5 min. The supernatant was discarded, and 500 µl of ROSE buffer and 1 g of 0.5 mm zirconia/silica beads (BioSpec Products, Bartlesville, USA) were added. The samples were homogenized at 6500 × *g* for 40 s using a Precellys homogenizer (Bertin Technologies, France). The homogenate was incubated at 90 °C for 10 min, followed by centrifugation at 16,000 × *g* for 5 min. The supernatant was transferred to a Maxwell 16 LEV Plant DNA kit cartridge, which was then placed into the Maxwell 16 robot (Promega, Madison, USA), where DNA was extracted automatically in accordance with the plant DNA extraction protocol. The DNA products were eluted in 200 µl of TE buffer and analyzed with a NanoDrop 2000 spectrophotometer (Thermo Scientific, Massachusetts, USA).

### NaOH-based DNA extraction

The DNA extraction from stool samples was conducted using a NaOH-based method as described by Shao et al. with modifications [24]. Briefly, 500 mg of faecal sample was mixed with 1 ml of phosphate-buffered saline (PBS) and vortexed for 15 s at 3000 rpm. Then, 1.5 ml of NaOH (0.2 mol/L) was added to the mixture and incubated at 95 °C for 10 min. After centrifugation at 3000 × *g* for 5 min, the upper liquid phase was diluted with 0.2 mol/L Tris–HCl at a 1:1 ratio. The Tris–HCl-neutralized crude extract was centrifuged at 16,000 × *g* for 5 min and 400 µl of the supernatant was used for DNA isolation with a QIAquick PCR Purification kit (QIAGEN, Hilden, Germany) and a DNA Clean & Concentrator-25 kit (Zymo Research, Irvine, CA, USA) following the manufacturer’s instructions. The final DNA products were eluted in 50 µl of TE buffer and analyzed using a NanoDrop 2000 spectrophotometer (Thermo Scientific, Massachusetts, USA).

### *Sm1-7*-qPCR

The DNA extracted from faecal samples was amplified using a real-time PCR system targeting a 121 bp sequence from *S. mansoni* (GenBank accession number M61098) [17, 25]. For DNA samples extracted with the Maxwell kit, the concentration was adjusted to 40 ng/µl if it exceeded 40 ng/µl, while no concentration adjustment was made for samples extracted using the NaOH-based method. Each sample was subjected to a 10 µl qPCR assay, including 1 µl of ddH_2_O, 5 µl of 2 × GoTaq Probe qPCR Master Mix (Promega, Madison, USA), 1 µl of 2 µmol/L forward primer (5′-CTG AAT CCG ACC AAC CGT TC-3′) (IDT, Singapore), 1 µl of 2 µmol/L reverse primer (5′-CCA CGC TCT CGC AAA TAA TCT-3′) (IDT, Singapore), 1 µl of 2.5 µmol/L FAM probe (5′-/56-FAM/ TCC GAA ACC / ZEN/ ACT GGA CGG ATT TTT ATG AT / 3IABkFQ/-3′) (IDT, Singapore) and 1 µl of template DNA. The qPCR assays were conducted on a QuantStudio 5 system (Thermo Scientific, Massachusetts, USA) with the following cycling conditions: 50 °C for 2 min; 95 °C for 2 min; and 40 cycles of denaturation at 95 °C for 30 s and annealing and extension at 60 °C for 60 s. A standard curve was established by testing a tenfold serial dilution of purified *S. mansoni* egg DNA (1 ng/μl–1 fg/μl). A total of 482 stool samples were analyzed in triplicate, with positive (0.1 ng/µl *S. mansoni* egg DNA) and no template controls included in each run. Samples with qPCR Ct values below the cut-off value from the standard curve were considered positive.

### Statistical analysis

The statistical analysis was performed using GraphPad Prism version 10.2.1 (GraphPad Software, San Diego, USA). Differences in DNA concentrations and Ct values were compared using a Wilcoxon signed-rank test, a paired *t*-test, or a Kruskal-Wallis test. The diagnostic performance [sensitivity, negative predictive value (NPV)] of the KK and different copro-DNA-qPCR systems was assessed using a composite reference where samples positive by either the KK method or qPCR assays were considered true positives. Sensitivities of different diagnostic methods were compared using McNemar’s chi-square (*χ*^2^_m_) test. The positivity rates of *S. mansoni* were evaluated for each diagnostic method. Differences in positivity rates between the different diagnostic methods and the composite reference were assessed using McNemar’s test (https://www.graphpad.com/quickcalcs/mcnemar1/). The Kappa coefficient (ƙ) was used to estimate the agreement between the different diagnostic methods with the combined reference (https://www.graphpad.com/quickcalcs/kappa1/). In addition, correlations between the faecal egg burden (expressed as EPG) and Ct values were calculated using Spearman’ s rank correlation co-efficient. *S. mansoni* prevalence across different endemic villages, sexes, and age groups was compared using a Chi-square (*χ*^2^) test. A *P*-value less than 0.05 was considered statistically significant.

## Results

### Standard curve

A standard curve was created to establish the correlation between the *S. mansoni* egg DNA inputs and *Sm1-7*-qPCR Ct values. The slope of the curve was -3.4881, indicating an assay efficiency of 93.5% based on the equation: E = (10^(−1/slope)^−1) × 100%. The goodness of fit for *Sm1-7-*qPCR was evaluated using the R^2^ value, which was 0.992 (Additional file 2: Fig. S1). The cut-off value was determined as 33.63, corresponding to an input of 1 fg *S. mansoni* genomic DNA. Samples with a mean Ct equal to or below 33.63 were considered positive, while those above and with no Ct value detected were determined as negative.

### Comparison of two DNA clean kits to be incorporated into the NaOH-based DNA extraction

To determine an effective DNA cleaning method that can be integrated into the NaOH-based DNA extraction, two commercial kits, the QIAquick PCR Purification kit and the Zymo DNA Clean & Concentrator-25 kit, were compared. A total of 15 faecal samples were used for the comparison, including 10 samples from *S. mansoni* endemic areas and 5 samples from a non-endemic area. Table 1 shows DNA concentration after purification and the results of *Sm1-7*-qPCR performed on both crude and kit-purified DNA products. The median DNA concentration for samples purified by the Zymo DNA Clean & Concentrator-25 kit was approximately 4 times higher than that of samples isolated using the QIAquick PCR Purification kit (*P* < 0.0001*,* Wilcoxon test). For samples from endemic areas, 6 out of 10 tested positive for *Sm1-7-*qPCR on crude DNA extracts. However, all 10 samples tested positive for *Sm1-7-*qPCR after purification with the kits*.* The mean Ct value from the samples purified with the Zymo kit was significantly lower than that isolated with the QIAGEN kit (*P* = 0.0005, paired *t*-test), resulting in a mean ΔCt value (Ct (Z)–Ct (Q)) of –3.696. Both DNA concentration and Ct value analyses indicate a significantly higher DNA recovery efficacy for the Zymo kit compared to the QIAGEN kit. For samples from a non-endemic area, no positive results were detected with *Sm1-7-*qPCR on both crude and kit-purified DNA products. Consequently, the Zymo DNA Clean & Concentrator-25 kit was further incorporated into the NaOH-based method.

**Table 1:** Results of copro-DNA-*Sm1*-*7-*qPCR conducted on NaOH-extracted DNA products

Sample source	KK (*Schistosoma mansoni* EPG)	Conc (Q) ng/µl	Conc (Z) ng/µl	Ct (C)	Ct (Q)	Ct (Z)	∆Ct
Schistosomiasis endemic area	0.0	10.0	17.2	25.205	29.027	21.520	−7.506
	15.9	26.9	63.6	Neg	22.807	18.410	−4.397
	51.6	12.4	20.0	21.403	17.712	16.565	−1.147
	170.6	49.2	186.5	Neg	24.486	22.256	−2.229
	71.4	42.3	137.0	22.341	20.571	13.201	−7.370
	31.7	70.1	209.0	16.654	13.761	11.390	−2.372
	131.0	18.9	100.0	19.417	16.176	13.398	−2.779
	1059.5	21.6	132.0	Neg	21.380	17.734	−3.646
	123.0	45.2	218.0	19.528	16.279	14.858	−1.420
	4.0	16.4	37.5	Neg	23.857	19.759	−4.098
Schistosomiasis non-endemic area	0.0	33.2	110.0	Neg	Neg	Neg	N/A
	0.0	14.8	43.7	Neg	Neg	Neg	N/A
	0.0	81.9	190.0	Neg	Neg	Neg	N/A
	0.0	98.5	287.0	Neg	Neg	Neg	N/A
	0.0	115.1	339.0	Neg	Neg	Neg	N/A

*KK* Kato-Katz, *EPG* Eggs per gram of faeces, *Conc (Q)* Concentration of DNA extracted using the QIAquick PCR Purification kit, *Conc (Z)* Concentration of DNA purified with the Zymo DNA Clean & Concentrator-25 kit. *Ct (C), Ct (Q)* and *Ct (Z)*: Ct values for testing crude DNA extracts, DNA purified using the QIAquick PCR Purification kit, and DNA purified using the Zymo DNA Clean & Concentrator-25 kit, respectively. ∆Ct = Ct (Z)–Ct (Q). *Neg* Negative, *N/A* Not applicable

### Development and comparison of two copro-DNA-qPCR systems for the detection of *S. mansoni* infection

**Fig. 2:**
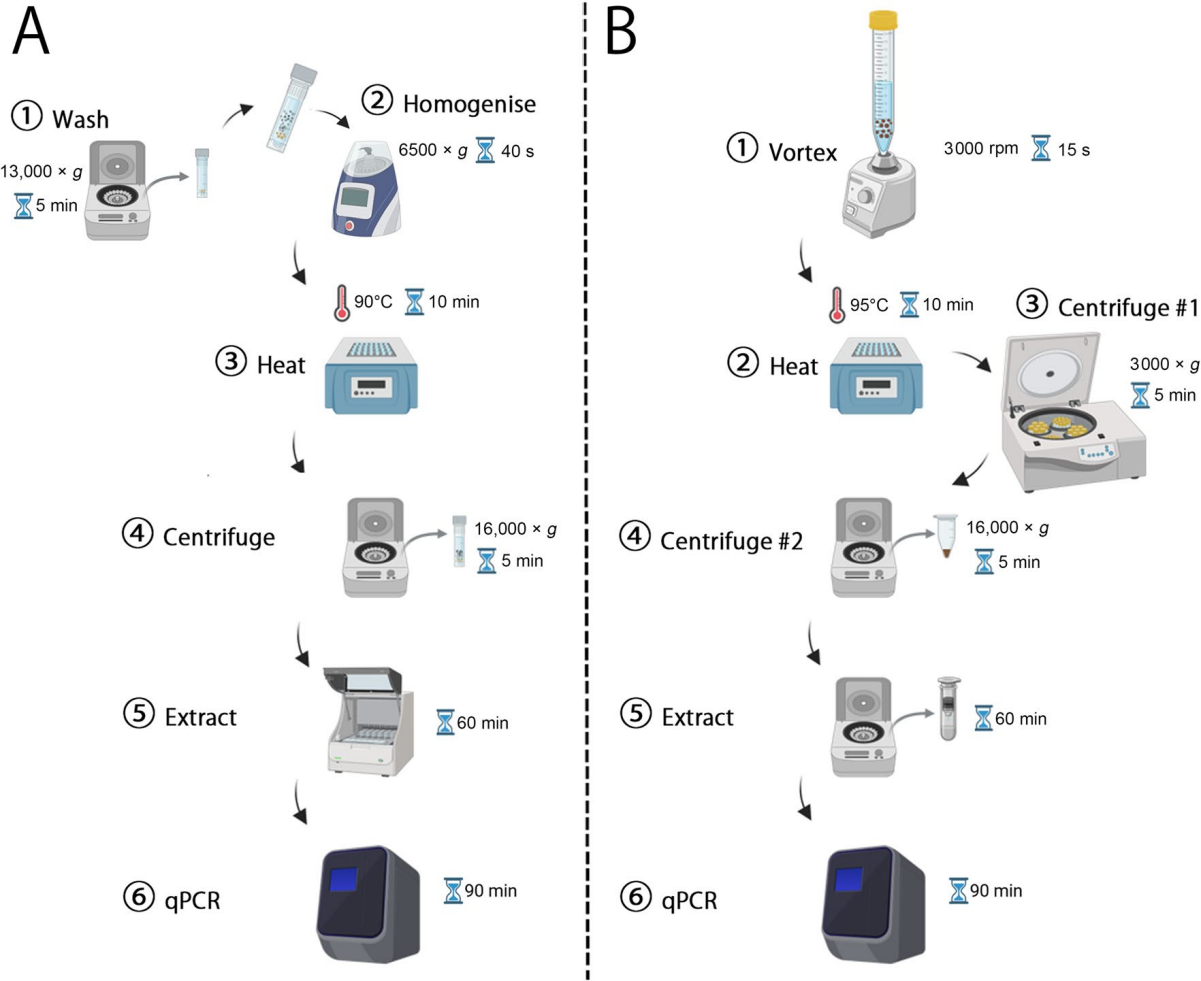
Workflows for the two copro-DNA-qPCR systems for detecting *Schistosoma mansoni* infection. **A** Maxwell kit-based copro-DNA-qPCR system (designated as *Sm1*-*7*-qPCR-S1): Faecal samples were washed once with ddH_2_O, homogenized using a Precellys homogenizer after embedding with zirconia/silica beads. Following a heat step on a heat block (90 °C, 10 min), the samples were centrifuged to obtain supernatant, which was further utilized for DNA extraction using the Maxwell 16 LEV Plant DNA kit on a Maxwell 16 instrument. **B** NaOH-based copro-DNA-qPCR system (designated as *Sm1*-*7*-qPCR-S2): Faecal samples were suspended in 1 × PBS (1 ml) and 0.2 mol/L NaOH (1.5 ml), heated at 95 °C for 10 min, neutralized with 0.2 mol/L Tris–HCl, and subjected to a two-step centrifugation. The resulting supernatants were transferred for DNA extraction using a DNA Clean & Concentrator-25 kit (Zymo Research). The DNA samples extracted from the two copro-DNA approaches were tested with the *Sm1-7*-qPCR assay on a QuantStudio 5 system. Figure created using BioRender (https://biorender.com/)

**Fig. 3:**
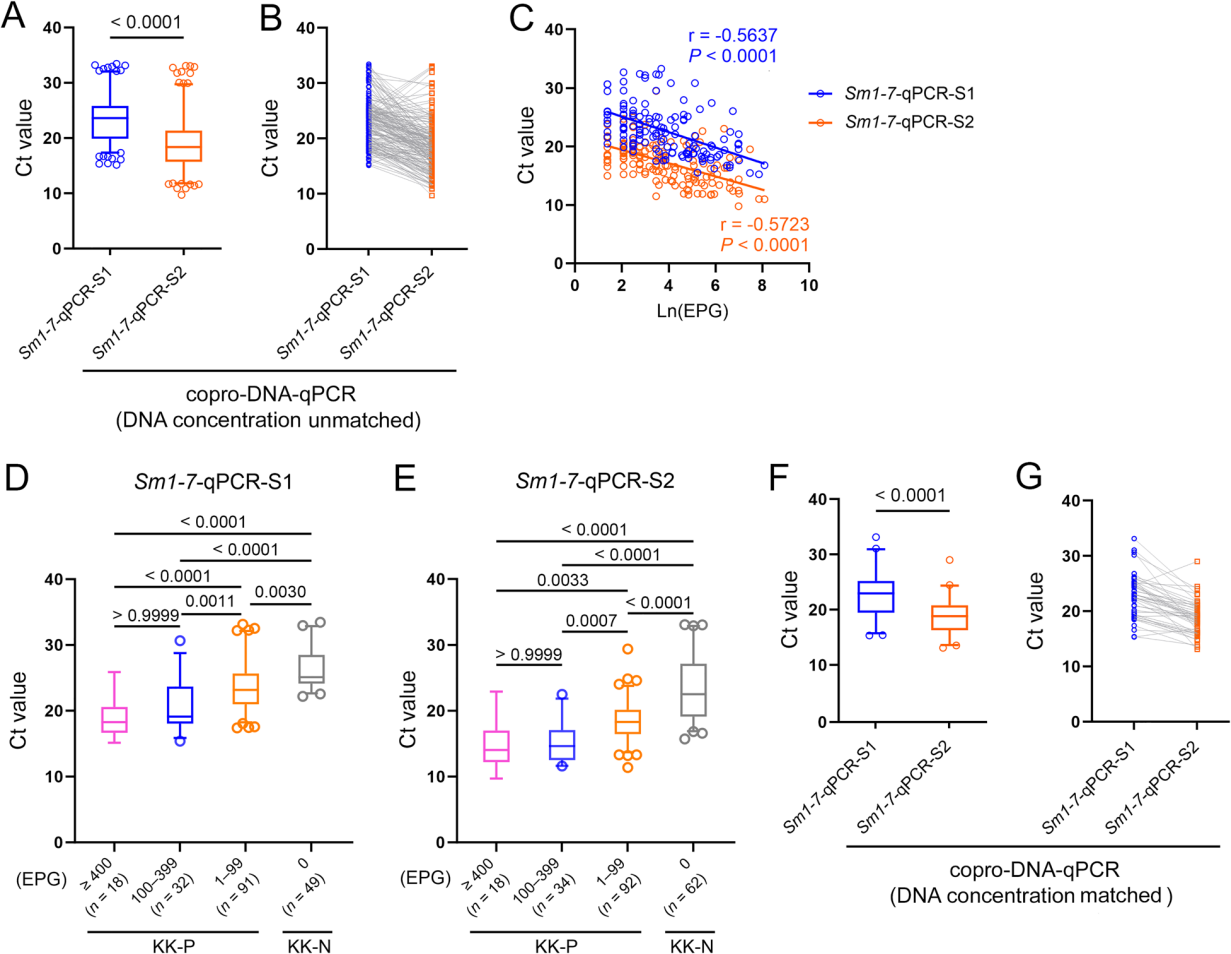
Comparison of results between *Sm1*-*7*-qPCR-S1 and *Sm1*-*7*-qPCR-S2. **A** Comparison of Ct values for DNA samples (*n* = 179) identified as positive by both *Sm1*-*7*-qPCR systems (*P* < 0.0001, Wilcoxon signed-rank test). **B** Differences in Ct values between the two systems are shown for the 179 positive samples. **C** Spearman’s rank correlation was used to measure the strength and direction of association between the faecal egg burden and Ct values from *Sm1*-*7*-qPCR systems. **D–E** Comparison of Ct values from KK-negative and different KK-positive subgroups stratified by different infection intensities for *Sm1*-*7*-qPCR-S1 **D** and *Sm1*-*7*-qPCR-S2 **E**. *P* values were calculated using a Kruskal-Wallis test. **F** Comparison of Ct values of *Sm1*-*7*-qPCR assays on testing concentration matched samples (*n* = 45) randomly selected from the 179 positive samples (*P* < 0.0001, Wilcoxon signed-rank test). **G** Differences in Ct values between the two systems are shown for the randomly selected concentration matched samples (*n* = 45). In Panels **A**, **D**, **E**, and **F**, the boxes represent the interquartile range of the data, while the lines across the boxes indicate the median values. The hash marks positioned below and above the boxes represent the 5th and 95th percentiles for each group, respectively. *EPG* Eggs per gram of faeces, *KK-P* Kato-Katz-positive, *KK-N* Kato-Katz-negative

We developed two copro-DNA-qPCR systems for detecting *S. mansoni* infection: a Maxwell kit-based system (*Sm1*-*7*-qPCR-S1) and a NaOH-based system (*Sm1*-*7*-qPCR-S2) (Fig. 2). Both systems were used to test identical faecal samples collected from schistosomiasis-endemic and non-endemic villages in Uganda (*n* = 433 and *n* = 49, respectively) (Additional file 1: Table S1). The median concentration of DNA purified by the NaOH-based method (96.10 ng/µl) was higher than that of the Maxwell kit-based method (69.25 ng/µl) (*P* < 0.0001, Wilcoxon test) (Additional file 2: Fig. S2A). There was a significant correlation in DNA concentration between the two systems (r = 0.3748, *P* < 0.0001) (Additional file 2: Fig. S2B). In the analysis of samples from endemic areas, the KK, *Sm1-7*-qPCR-S1, and *Sm1*-*7*-qPCR-S2 identified 159 (36.7%), 190 (43.9%), and 206 (47.6%) samples as positive, respectively (Table 2). *Sm1*-*7*-qPCR-S1 recorded minimum and maximum Ct values of 15.14 and 33.46, respectively, with a median of 23.59, whereas *Sm1*-*7*-qPCR-S2 showed minimum and maximum Ct values of 9.70 and 33.08, respectively, with a median of 18.38 (Table 2). For the samples identified as positive by both *Sm1-7*-qPCR-S1 and *Sm1*-*7*-qPCR-S2 (*n* = 179), the Ct values obtained from *Sm1*-*7*-qPCR-S2 were significantly lower than those from *Sm1*-*7*-qPCR-S1 (*P* < 0.0001, Wilcoxon test) (Fig. 3A). A total of 158 samples exhibited lower Ct values in *Sm1*-*7*-qPCR-S2 compared to *Sm1-7*-qPCR-S1 (Fig. 3B). There were significant inverse correlations between faecal egg burden and Ct values from *Sm1-7*-qPCR in both systems (*Sm1-7*-qPCR-S1, r = −0.5637, *P* < 0.0001; *Sm1*-*7*-qPCR-S2, r = −0.5723, *P* < 0.0001) (Fig. 3C). To further investigate the relationship between egg burden and qPCR outcomes, KK-positive individuals were classified based on infection intensity as light (1–99 EPG), moderate (100–399 EPG) and heavy (≥ 400 EPG) according to WHO criteria [26]. In both *Sm1*-*7*-qPCR systems, the median Ct values obtained from all KK-positive subgroups were significantly lower compared to that from KK-negative group (Fig. 3D, E, Additional file 1: Table S2). Among the KK-positive individuals, there was no significant difference in Ct value between subgroups with heavy and moderate infection intensities. However, both subgroups showed significantly lower Ct values compared to the subgroup with light infections (Fig. 3D, E, Additional file 1: Table S2).

**Table 2:** Prevalence of *Schistosoma mansoni* and descriptive statistics for the positive cases identified by the different diagnostic methods

Method	Positive cases/total	Positivity rate (%)	EPG (KK)/Ct-values (qPCR)		
			Median	Minimum	Maximum
KK	159/433	36.7	31.75	3.97	3246.0
*Sm1*-*7*-qPCR-S1	190/433	43.9	23.59	15.14	33.46
*Sm1*-*7*-qPCR-S2	206/433	47.6	18.38	9.70	33.08

*EPG* Eggs per gram of faeces, *KK* Kato-Katz

To assess the impact of DNA concentration on copro-DNA-qPCR results, 45 DNA samples extracted using the NaOH-based method and purified with the Zymo kit were randomly selected. These samples were diluted to match the concentration of the corresponding DNA samples tested in *Sm1*-*7*-qPCR-S1 and re-tested using the *Sm1*-*7*-qPCR assay. The median Ct value from the re-tested assay was significantly lower than that from *Sm1*-*7*-qPCR-S1 (*P* < 0.0001, Wilcoxon test) (Fig. 3F), with 41 samples showing a lower Ct value in *Sm1-7*-qPCR-S2 compared to *Sm1*-*7*-qPCR-S1 (Fig. 3G). For samples that tested positive for *S. mansoni* by the KK and/or *Sm1*-*7*-qPCR-S1 but negative by *Sm1*-*7*-qPCR-S2 (*n* = 23), DNA samples purified using the Zymo kit were re-tested after being diluted 10-fold with ddH_2_O. Upon re-testing, 3 samples showed positive results, indicating amplification failure caused by the presence of inhibitors in certain samples extracted using the NaOH-based method when tested without dilution. This occurred at a rate of 1.3%, based on the confirmation of 229 samples as true positive according to the composite reference. Out of 49 samples from a non-endemic area all KK negative for *S. mansoni* infection, one sample tested positive (Ct = 33.196) in *Sm1-7*-qPCR-S1 (Additional file 1: Table S1).

### Performance of different diagnostic methods calibrated with the composite reference

The sensitivity of the KK method, *Sm1-7*-qPCR-S1, and *Sm1-7*-qPCR-S2 was 69.4%, 83.0%, and 90.0%, respectively, when compared to the composite reference. The sensitivity of *Sm1-7*-qPCR-S1 and *Sm1*-*7*-qPCR-S2 was significantly higher than that of the KK method (*P* = 0.0002 and *P* < 0.0001, respectively, McNemar’s test). Notably, *Sm1*-*7*-qPCR-S2 had greater sensitivity than *Sm1*-*7*-qPCR-S1 (*P* = 0.0150, McNemar’s test) (Table 3). The composite reference recorded an *S. mansoni* prevalence rate of 52.9%. There were significant differences in *S. mansoni* prevalence determined by the different methods compared to the composite reference (*P* < 0.0001) (Table 3). The KK method had substantial agreement with the reference (*ƙ* = 0.682), while both *Sm1*-*7*-qPCR systems exhibited almost perfect agreement with the reference (*ƙ* = 0.821 and 0.894, respectively) (Table 3). By analysing all tested samples (*n* = 482), the KK method demonstrated substantial agreement with both *Sm1*-*7*-qPCR systems (*ƙ* = 0.696 and 0.664, respectively), while *Sm1-7*-qPCR-S1 exhibited almost perfect agreement with *Sm1-7*-qPCR-S2 (*ƙ* = 0.833) (Additional file 1: Table S3).

**Table 3:** Sensitivity, negative predictive value (NPV), prevalence and Kappa coefficient of the different diagnostic methods calibrated to a composite reference

		Reference test*		% Sensitivity ^#^ (95% *CI*)	% NPV (95% *CI*)	Prevalence % (*P*, McNemar’s test)	Kappa imdex (95% *CI*)
		+	−				
KK	+	159	0	69.4 (63.2–75.0)	74.5 (69.0–79.3)	36.7 (< 0.0001)	0.682 (0.617–0.746)
	−	70	204				
*Sm1-7*-qPCR-S1	+	190	0	83.0 (77.6–87.3)	84.0 (78.8–88.0)	43.9 (< 0.0001)	0.821 (0.768–0.874)
	−	39	204				
*Sm1-7*-qPCR-S2	+	206	0	90.0 (85.4–93.2)	89.9 (85.3–93.2)	47.6 (< 0.0001)	0.894 (0.852–0.936)
	−	23	204				

*CI* Confidence interval, *KK* Kato-Katz, *NPV* Negative predictive value. *KK and copro-DNA-qPCR results were used as a composite reference where any positives from either the KK or qPCR assays were considered true positives. ^#^Statistical differences in sensitivity between different diagnostic methods were assessed using McNemar’s test: *Sm1-7*-qPCR-S1 *vs* KK (*χ*^2^_m_ = 13.433, *P* = 0.0002); *Sm1-7*-qPCR-S2 *vs* KK (*χ*^2^_m_ = 27.481, *P* < 0.0001); *Sm1-7*-qPCR-S1 *vs* *Sm1-7*-qPCR-S2 (*χ*^2^ _m_ = 5.921, *P* = 0.0150)

### Performance of the copro-DNA-qPCR systems stratified by infection status and intensity

Out of the 159 participants tested positive for *S. mansoni* using the KK method, 107 (67.3%) had light infections, 34 (21.4%) had moderate infections, and 18 (11.3%) had heavy infections. Both copro-DNA-qPCR systems showed absolute sensitivity for individuals with heavy infections. *Sm1-7*-qPCR-S1 had a sensitivity of 94.1% for moderate infections, while *Sm1-7*-qPCR-S2 exhibited 100% sensitivity for this category. Both copro-DNA-qPCR systems displayed a similar sensitivity (~ 85.5%) in detecting individuals with light infections (Table 4). Additionally, among the 274 participants who tested negative for *S. mansoni* using the KK technique, *Sm1-7*-qPCR-S2 recorded a higher positivity rate compared to *Sm1-7*-qPCR-S1 (22.6% *vs* 17.9%, *P* = 0.0259).

**Table 4:** Sensitivities and positivity rates of the two copro-DNA-qPCR systems stratified by infection status and intensity

Method	Heavy (≥ 400 EPG)^#^ (*n* = 18)	Moderate (100–399 EPG) ^#^ (*n* = 34)	Light (1–99 EPG) ^#^ (*n* = 107)	Negative (0 EPG) (*n* = 274)
	Sensitivity % (95% *CI*)			Positivity % (95% *CI*)
*Sm1-7*-qPCR-S1	100 (82.4–100)	94.1 (80.9–99.0)	85.0 (77.1–90.6)	17.9 (13.8–22.9)
*Sm1-7*-qPCR-S2	100 (82.4–100)	100 (89.8–100)	86.0 (78.2–91.3)	22.6 (18.1–27.9)
Differences (*χ*^2^_m_, *P*)^*^	N/A	0.500, 0.4795	0.000, 1.000	4.966, 0.0259

*CI* Confidence interval, *EPG* Eggs per gram of faeces, *N/A* Not applicable. ^#^Infection intensity stratified according to the WHO criteria using the KK technique for the diagnosis of schistosomiasis (26). ^*^Statistical differences in sensitivity and positivity rate were assessed using McNemar’s test

### *S. mansoni* prevalence across different endemic villages, sexes, and age groups

We further analyzed the prevalence of *S. mansoni* in the five endemic villages, based on the results of different diagnostic methods and the composite reference (Additional file 1: Table S4). According to the village-level prevalence derived from the combined gold standard, Bugoto exhibited the highest prevalence at 66.3%, followed by Bwondha (64.5%), Musubi (62.6%), and Bukizibu (37.4%), with Igeyero having the lowest prevalence (18.0%). Males generally exhibited a slightly higher prevalence rate than females; however, a significant difference was only observed in *Sm1-7*-qPCR-S1 (*χ*^2^ = 5.869, *P* = 0.0150). There was no significant difference in prevalence between the two age groups (5–14 years old *vs* > 14 years old) across all the diagnostic methods (Additional file 1: Table S4).

## Discussion

In this study, we introduced two copro-DNA-qPCR systems for the molecular diagnosis of schistosomiasis mansoni. The positivity rates for *S. mansoni* infection were higher with *Sm1-7*-qPCR-S1 (43.9%) and *Sm1-7*-qPCR-S2 (47.6%) compared to the KK method (36.7%), indicating the superior sensitivity of the qPCR systems over traditional parasitological methods. Previous studies have also reported increased positivity rates with NAAT-based molecular approaches. For instance, Mesquita et al. found a 16.5% *S. mansoni* positivity rate using PCR targeting the *Sm1-7* gene, compared to 5.5% with the KK method [27]. Similarly, in a study carried out in Kirinyaga County, Kenya, qPCR detected a 72.8% positivity rate for *S. mansoni*, which is about 2.2 times higher than that determined by the parasitological technique (32.8%) [28]. In addition, the parasite load of the examined participants was categorized according to the EPG values identified by the KK [26]. In both *Sm1-7*-qPCR systems, the median Ct values gradually increased from the KK-positive subgroups with heavy, moderate, and light infections to the KK-negative group (Fig. 3D, E). Among the KK-positive individuals, most negative qPCR results were observed in those with light infections (Table 4). These findings demonstrate the effectiveness of molecular techniques as reliable options for parasite diagnosis.

The evaluation of novel diagnostics faces challenges due to the absence of a definitive gold standard. To address this issue, a composite reference combining outcomes of different tests, such as parasitological, circulating antigen assays, and molecular techniques, was frequently used to provide a more reliable classification of true positive cases [29–31]. In this study, we integrated the outcomes of KK and qPCR assays as a composite reference, which establishes a solid basis for the assessment of the new systems, mitigating the limitations of relying on a single flawed gold standard, such as the KK method [32]. In comparison to the composite reference, the sensitivity of *Sm1-7*-qPCR-S1 and *Sm1-7*-qPCR-S2 showed a significant difference compared to that of the KK method (*P* = 0.0002 and *P* < 0.0001, respectively). Both *Sm1-7*-qPCR systems exhibited almost perfect agreement with the composite reference, confirming their reliability in disease diagnosis. Consistent results indicating a high level of concordance between qPCR and the composite reference standard were also reported in previous studies. Notably, *Sm1-7*-qPCR-S2 exhibited higher sensitivity than *Sm1-7*-qPCR-S1 (*P* = 0.015). This increased sensitivity may be attributed to the direct testing of undiluted DNA samples in *Sm1-7*-qPCR-S2, whereas DNA concentration adjustment was made in *Sm1-7*-qPCR-S1 to minimize the risk of inhibitors.

Molecular diagnosis often requires nucleic acid extraction using commercial DNA or RNA extraction kits [13, 33, 34]. The *Sm1-7*-qPCR-S1 system is more laboratory-intensive, requiring special equipment such as a homogenizer and a Maxwell 16 robot. In pursuit of enhanced field applicability, *Sm1-7*-qPCR-S2 integrated the NaOH-based DNA extraction method with a simple DNA clean-up procedure. The system can become more field-friendly by replacing the QuantStudio 5 with a portable qPCR machine or by integrating an isothermal assay, such as loop-mediated isothermal amplification (LAMP) [35–37], lateral flow recombinase polymerase amplification (LF-RPA) assays [38, 39] and RPA-CRISPR assays [40, 41]. Notably, when testing 45 matched DNA samples at identical concentrations, the Ct values from *Sm1*-*7*-qPCR-S2 were significantly lower than those from *Sm1*-*7*-qPCR-S1, indicating a higher abundance of parasite-derived DNA in samples extracted through the NaOH-based method. This could be attributed to a large amount of faecal sample as starting material and the effective lysis action of NaOH at high temperature, a process which could facilitate the breakdown of eggshells and cell membranes, as well as the denaturation of biomolecules such as DNA and proteins [42]. Given that parasite eggs are not evenly distributed in faecal samples [43], a greater amount of stool as initial input may lead to more effective enrichment of egg-derived DNA in the final DNA extracts. The NaOH-based DNA extraction method has been incorporated with RPA-CRISPR/Cas12a assay for detecting *Echinococcus granulosus* infection, demonstrating acceptable sensitivity (68.0%) and absolute specificity (100%) [24]. The study did not include a clean-up and concentration step for the extracted crude DNA, which could have affected the sensitivity [24]. Similarly, the *Sm1*-*7*-qPCR only achieved 60.0% sensitivity when testing crude DNA extracts from samples collected in an endemic area (Table 1), indicating significant PCR inhibition as observed in previous studies [44, 45]. PCR inhibition is a well-recognized limitation of stool-based molecular diagnostics due to the presence of complex inhibitory substances in faecal matrices [44, 45]. In this study, while most samples performed reliably, a small subset of samples that were initially negative by *Sm1-7*-qPCR-S2 became positive upon re-testing at a tenfold dilution, confirming the presence of PCR inhibitors in undiluted DNA extracts. Nevertheless, amplification failure due to inhibition only affected a small proportion of infected individuals (1.3%, 3/229), indicating that the NaOH-based extraction method combined with a DNA clean-up step provides an effective balance between analytical sensitivity and assay robustness. The cost of *Sm1-7*-qPCR-S1 is higher than that of *Sm1*-*7*-qPCR-S2; however, it is less labor-intensive due to the automatic DNA extraction by a Maxwell robot. In addition, both systems have similar time efficiency in DNA extraction.

According to the established standard curve, samples were considered positive for qPCR when the Ct value was 33.63 or lower, which corresponds to the detection of 1 fg/μl of *S. mansoni* genomic DNA. This finding is consistent with the results of a recent study [17]. The ability to quantify DNA through qPCR facilitates the assessment of parasite load in infected individuals. In this study, significant inverse correlations were observed between the faecal egg burden and Ct values from *Sm1-7*-qPCR in both systems, demonstrating a comparable association strength (r = −0.5637 and −0.5723, respectively), consistent with previous studies [17, 46]. For example, a negative correlation (r = −0.695) between *S. mansoni* egg loads and Ct values in a qPCR assay has been previously reported [46]. Nevertheless, several factors may affect the strength of these correlations. For instance, qPCR was performed solely on the first day stool sample, while the KK was performed on two stool samples collected on two consecutive days. Additionally, the overall infection intensity, dietary intake, the texture of stool samples, as well as the presence/concentration of inhibitors in the extracted DNA samples may also affect the coefficient. There are several ways to improve the accuracy of the coefficient. These include conducting copro-DNA-*Sm1*-*7*-qPCR on two stool samples collected from each individual, using inhibitor-tolerant qPCR detection methods, and normalizing *Sm1-7*-qPCR Ct values to a spike-in internal control, such as phocine herpesvirus-1 [47].

The WHO’s guidelines for schistosomiasis control [48] recommend that new diagnostic options for schistosomiasis should be affordable, easy-to-use, and require minimal training for health professionals conducting the tests. The cost of DNA extraction in *Sm1-7*-qPCR-S1 is approximately USD6, primarily due to the Maxwell 16 LEV Plant DNA kit. In contrast, the cost for DNA extraction in *Sm1-7*-qPCR-S2 is about USD3.0, mainly attributed to the Zymo kit, as the NaOH lysis procedure itself is relatively inexpensive [37]. However, it is possible to replace the Zymo kit with a more economical alternative. The NaOH-based DNA extraction is more practical for field applications compared to the Maxwell kit-based method, which relies on a Maxwell 16 robot. Although both methods require additional equipment such as a centrifuge and a heat block, these instruments are becoming increasingly accessible for deployment in endemic sentinel sites as regional infrastructure and economies develop. Overall, a comparative evaluation of the costs, diagnostic performance, and equipment requirements of the two *Sm1-7*-qPCR systems suggests that *Sm1-7*-qPCR-S2 is a more promising tool for schistosomiasis diagnosis. However, further refinement of the system is necessary to meet the WHO’s ASSURE criteria [49], and field studies are essential to validate the system’s effectiveness in practical settings following optimization. Moreover, adapting the NaOH-based copro-DNA-qPCR system to detect other pathogens, such as soil-transmitted helminths commonly found in schistosomiasis-endemic areas, would greatly benefit helminth control efforts. Nevertheless, optimization and validation remain essential for implementing such a system to detect other pathogens. For example, it has been proposed that incorporating a bead-beating procedure during faecal DNA extraction is critical for the molecular detection of *Trichuris trichiura* [50].

The major strengths of this study include the relatively large sample size and the fact that the samples were collected from both schistosomiasis endemic and non-endemic areas in Uganda. However, the study has several limitations. First, similar to other studies reporting qPCR-based NAATs, there is a lack of standardized Ct values for *Sm1-7*-qPCR for comparison with analogous studies on schistosomiasis diagnosis across different endemic areas. Second, the employment of a combined standard reference may not effectively reduce bias in sensitivity estimates of *Sm1-7*-qPCR-based molecular diagnostics. We used the KK along with the *Sm1-7*-qPCR systems as a combined reference, but KK is recognized as a flawed test. Therefore, our results should be interpreted cautiously due to the limitations associated with the composite reference [51]. Third, neither of the *Sm1-7*-qPCR systems meets the WHO ASSURED criteria for diagnostics in low- and middle-income countries (LMICs), highlighting the need for additional optimisation to adapt *Sm1-7*-qPCR-S2 to meet diagnostic field requirements in resource-limited settings. For example, the system can be further integrated with a portable, low-cost PCR device or field-friendly isothermal amplification techniques, such as LAMP and RPA, to facilitate its field compatibility. Fourth, molecular detection was only performed on stool aliquot from the first day’s collection. A more accurate comparison of different diagnostic methods may be achieved by conducting copro-DNA-*Sm1-7*-qPCR on two stool samples collected on two consecutive days. Finally, approximately 56 million women and girls in LMICs are at risk of female genital schistosomiasis (FGS), primarily associated with *S. haematobium* [52]. However, there is evidence indicating that *S. mansoni* also causes FGS and persistent vulvar itch [53, 54]. The utility of the *Sm1-7*-qPCR systems for detecting FGS caused by *S. mansoni* was not explored in this study and should be investigated in future research, given the significant impact of FGS as a public health issue in regions with active schistosomiasis transmission in Africa [55].

## Conclusions

This study demonstrates that both the Maxwell kit-based and NaOH-based DNA extraction approaches generate DNA suitable for reliable *Sm1-7*-qPCR amplification for the detection of *S. mansoni*. Both *Sm1-7*-qPCR systems outperform the KK method in identifying *S. mansoni* infections, with Ct values exhibiting significant inverse correlations with the faecal egg count. However, the NaOH-based method allows for the purification of DNA from a larger amount of faecal sample, resulting in a higher abundance of egg-derived DNA in the extracted products, thus showing superior diagnostic performance, reflected by higher sensitivity and lower Ct values. *Sm1-7*-qPCR-S2 also offers other advantages over *Sm1-7*-qPCR-S1, such as reduced cost and minimal equipment requirements. These characteristics position *Sm1-7*-qPCR-S2 as particularly well suited for epidemiological surveillance, monitoring of control interventions, and detection of low-intensity infections in elimination and post-elimination settings. With further validation and standardization, the *Sm1-7*-qPCR-S2 system could serve as a practical molecular diagnostic tool for integration into schistosomiasis surveillance frameworks in endemic regions, aligning with the WHO’s schistosomiasis elimination plan. This approach can also be adapted for diagnosing other helminthic parasites, aiding in the control of helminthiases.

## Supplementary Information

Supplementary Material 1.

Supplementary Material 2.

## Data Availability

All data supporting the findings of this study are available within the paper and Supplementary Information.

## Abbreviations

*CI*: Confidence interval
cPCR: Conventional PCR
EPG: Eggs per gram of faeces
FGS: Female genital schistosomiasis
KK: Kato-katz
LAMP: Loop-mediated isothermal amplification
LF-RPA: Lateral flow recombinase polymerase amplification
LMICs: Low- and middle-income countries
NAATs: Nucleic acid amplification tests
NPV: Negative predictive value
NTDs: Neglected tropical diseases
POC-CCA: Point-of-care circulating cathodic antigen
qPCR: Quantitative real-time PCR
WHO: World Health Organization
