# Supplementary material for: Comparative evaluation of Sm1-7-qPCR systems incorporated with Maxwell kit-based and NaOH-based DNA extraction methods for the detection of Schistosoma mansoni infection
Source: Infect Dis Poverty. 2026 May 11;15:54. doi: 10.1186/s40249-026-01444-7 (PMC13159289; doi:10.1186/s40249-026-01444-7)
Supplement: Supplementary file 2 — Supplementary Material 2. [file 40249_2026_1444_MOESM2_ESM.docx]

Supplementary Information


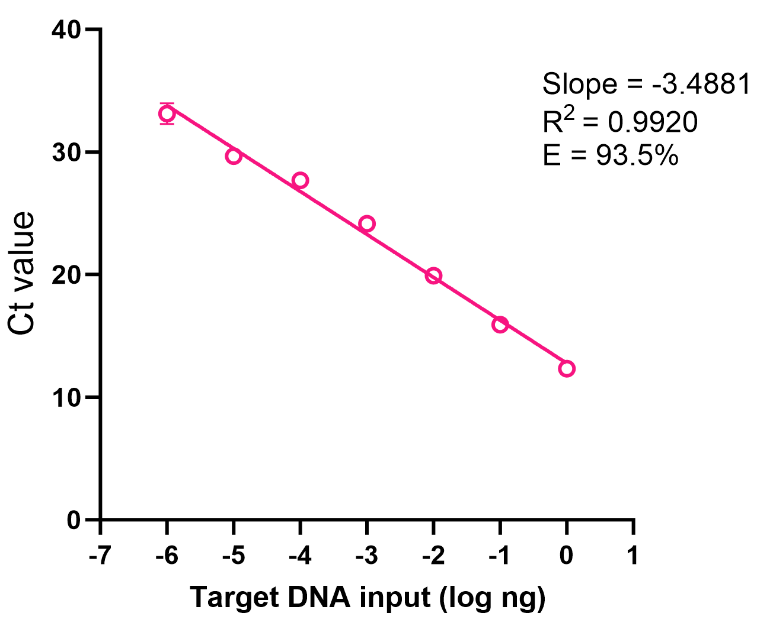


**Fig. S1** Graph representing the series of Schistosoma mansoni egg DNA inputs of the standard curve with Sm1-7-qPCR Ct values. All error bars are calculated based on results obtained from three replicates. R^2^ = coefficient of determination and E = reaction efficiency.


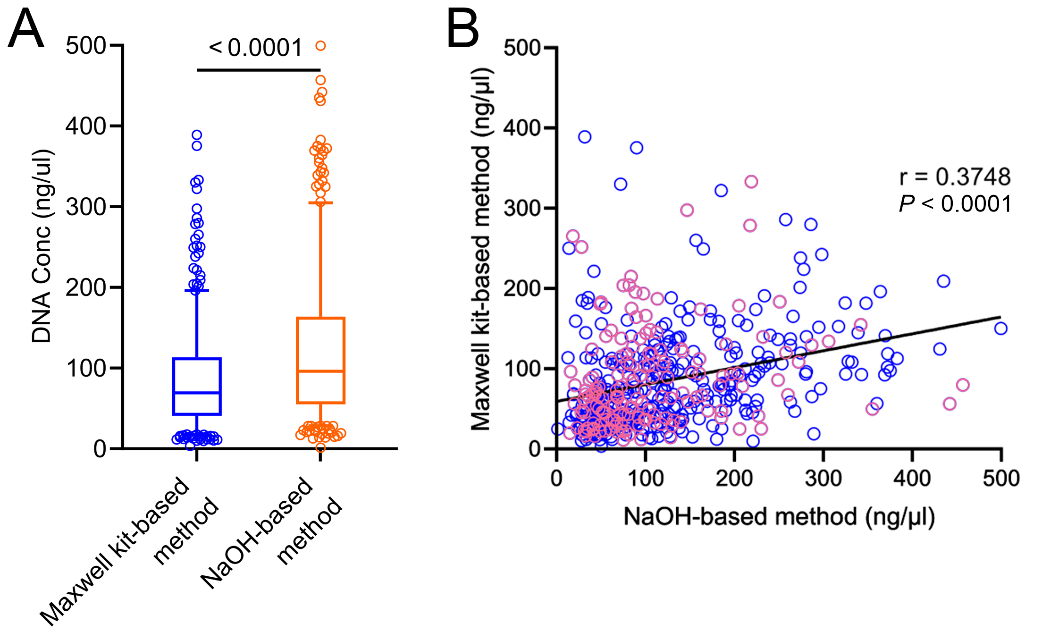


**Fig. S2** DNA concentration analysis. **A** Comparison of DNA concentration of samples (n = 482) extracted using Maxwell kit-based and NaOH-based methods (P < 0.0001, Wilcoxon signed-rank test). The boxes represent the interquartile range of the data, while the lines across the boxes indicate the median values. The hash marks positioned below and above the boxes represent the 5th and 95th percentiles for each group, respectively. **B** Correlation of DNA concentration between the two methods was measured using Spearman's rank correlation coefficient. Pink and blue cycles represent KK-positive and KK-negative samples, respectively.
